# A comparative study of 8-week complex training and resistance training on athletic performance of amateur futsal players

**DOI:** 10.3389/fphys.2024.1360440

**Published:** 2024-04-26

**Authors:** Yuan Zhai, Guoyang Qin

**Affiliations:** ^1^ Physical Education Department, Nanjing University of Aeronautics and Astronautics, Nanjing, China; ^2^ College of Physical Education, Shandong Normal University, Jinan, China

**Keywords:** performance enhancement, muscular strength, training efficacy, power, sprint

## Abstract

**Background:** Despite the acknowledged importance of resistance training (RT) in enhancing physical performance in futsal players., the comparative effectiveness of RT and complex training (CT) on both physical and technical performance in futsal players remains underexplored. This study aimed to compare the effects of RT vs. CT on physical and technical performance in amateur futsal players.

**Method:** Players from two amateur futsal teams were assigned to RT (one team of 16 players; 18 years) and CT (one team of 16 players; 18 years) to perform an 8-week intervention with two weekly sessions. The RT performed the squat and deadlift (6 sets of 6–10 repetitions at 75%–85% one-repetition maximum (1RM), while the CT performed the squat + squat jump and deadlift + high pull (3 sets of 4–6 + 10–12 repetitions at 75%–85% 1RM). Pre- and post-intervention assessments included the Futsal Special Performance Test (FSPT), repeated sprint ability (RSA), sprint decrement (Sdec), sprint times at 10-m (T10), 10–20-m (T10-20), and 20-m (T20), 1RM back squat (1RM BS), isometric mid-thigh pull (IMTP), and countermovement jump (CMJ).

**Results:** At baseline, no significant differences between groups were observed for any variable analyzed (*p* > 0.05). After 8 weeks, there were significant differences between CT vs. RT on FSPT (−10.8% vs. −3.4%; *p* < 0.05), T10 (−5.2% vs. −0.1%; *p* < 0.05), IMTP (7.8% vs. 5.1%; *p* < 0.05), and CMJ (10.2% vs. 4.5%; *p* < 0.05). On the other hand, no significant differences between CT vs. RT were observed for RSA (−2.0% vs. −1.2%; *p* > 0.05), Sdec (−7.6% vs. −3.5%; *p* > 0.05), T10-20 (−0.9% vs. −0.9%; *p* > 0.05), T20 (−1.8% vs. −1.7%; *p* > 0.05), and 1RM BS (5.7% vs. 4.5%; *p* > 0.05) after the training program. Both groups significantly improved FSPT, T20, 1RM BS, and IMTP, while only CT significantly improved RSA, Sdec, T10, and CMJ.

**Conclusion:** The results suggest that CT may be valuable for improving specific performance parameters in amateur futsal players, with some advantages over RT in enhancing strength and power. These findings support tailored training protocols for futsal players to optimize performance.

## 1 Introduction

Futsal is a multiple-sprint sport, with high-intensity movements dominating the majority of the game (Barbero-Alvarez et al., 2008; Dogramaci et al., 2011). The movement patterns and physical demands of futsal have been extensively studied. Studies have revealed that elite players perform approximately 400–500 high-intensity movements during a futsal match, primarily short accelerations, decelerations, sprints, and directional changes (Barbero-Alvarez et al., 2008; Makaje et al., 2012). These movements occur at regular intervals, typically every 7–9 s (Dogramaci et al., 2011). In a futsal match, sprint distances typically account for 5%–10% of the total distance, indicating the high physical demands of this challenging team sport (Barbero-Alvarez et al., 2008; Castagna et al., 2009; De Oliveira Bueno et al., 2014). However, A comparative analysis between elite and subelite players reveals significant differences in performance metrics. Elite futsal players demonstrated a higher number of sprints, longer sprinting durations, and covered more distance per sprint compared to subelite players (Dogramaci et al., 2011). In addition, a study involving elite futsal players found that the distance covered per minute, number of sprints, decelerations, and metabolic power are crucial physical factors that can differentiate the top elite futsal players (Ribeiro et al., 2020). These features can differentiate players at different levels and can be crucial physical qualities for achieving top performance in the game. Among these features, the capacity of players to consistently repeat high-intensity running without fatigue is a key determinant of successful performance in futsal (De Oliveira Bueno et al., 2014). Hence, emphasis should be placed on developing this particular capability (repeated-sprint ability, RSA) in training sessions.

Sports science and coaching have proposed various training methodologies (futsal injury prevention programs, visual feedback, high-intensity interval training and resistance training (RT)) to optimize the physical preparation of futsal athletes (Bayrakdaroğlu et al., 2022; Marques et al., 2022; Saryono et al., 2022; Gómez et al., 2023). RT is a typical training approach to enhance the maximal strength and power in athletes (Marques et al., 2019; Marques et al., 2022), ultimately aiming to improve RSA. However, RSA is a complex quality related to several factors, including anthropometry, endurance capacity (e.g., VO_2max_ and speed to reach VO_2max_), and neuromuscular performance (e.g., vertical jump, sprinting speed, and muscle strength) (Bishop et al., 2003; Brocherie et al., 2014; Buchheit and Mendez-Villanueva, 2014). Over the past decade, scientific research examining the impact of various RT programs on RSA has intensified, with several studies highlighting RT’s potential to enhance RSA (Edge et al., 2006; Paz-Franco et al., 2017a; Ramos-Campo et al., 2018; Torres-Torrelo et al., 2018). Despite these positive findings, further investigations reveal that RT, when applied as the sole training modality, may not uniformly improve all aspects of RSA in sports such as futsal and handball, particularly concerning average peak power or mean sprint time (RSAmean) (Hermassi et al., 2017; Torres-Torrelo et al., 2018). The effectiveness of RT in augmenting RSA, specifically RSAmean, appears to have its limitations. For example, Torres-Torrelo et al. (2018) demonstrated that while strength improvements (1RM) can enhance RSA, the benefits diminish with an increase in the number of sprints, suggesting that as the sprinting demand becomes more endurance-oriented, the aerobic system’s contribution might play a more significant role (Torres-Torrelo et al., 2018).

Complex training (CT) combines high-load RT and plyometric training within a single session. Recent studies have highlighted the efficacy of CT in improving sprint performance, jump height, and back squat 1RM in various sports (Qiao et al., 2022; Scott et al., 2023). It is thought that CT’s unique mix of heavy load resistance exercises followed by plyometric or speed exercises in the same session uses the post-activation potentiation (PAP) effect to improve sprint performance, while RSA was highly dependent on the sprint ability. Despite these promising findings, the application and impact of CT on RSA, specifically within the context of amateur futsal players, have not been thoroughly investigated.

Thus, this study aimed to compare the effects of CT vs. RT on physical and technical performance in amateur futsal players. It was hypothesized that both training interventions would improve physical and technical performance in amateur futsal players, with greater improvements observed after CT.

## 2 Materials and methods

### 2.1 Subjects

A minimum sample size of 24 participants was determined using GPower (version 3.1.9.7, Franz Faul, University of Kiel, Kiel, Germany), based on an alpha error probability (α) of 0.05, a power (1-β error probability) of 0.8, an effect size (ES) of 0.4, and including tests such as F-test and analysis of variance (ANOVA) for repeated measures and within-between interactions (Beck, 2013). For the study, 32 players from two amateur futsal teams within Jiangsu Province, China, were recruited, with a 20% anticipated dropout rate factored in to ensure an adequate sample size that compensates for potential participant attrition, aligning with the sample size requirements previously calculated. The recruitment phase spanned from August 1 to 1 September 2022, targeting individuals from collegiate backgrounds actively participating in regional competitions from March to August annually. The observational phase of the study was scheduled off-season, from October 1 to December 1, to preclude interference from official competitive engagements. This period of inactivity facilitated an undisturbed examination of the participants, who were stationed in Nanjing, Jiangsu Province, during the study duration. A detailed explanation was provided to the participants regarding the aims, benefits, and risks associated with the investigation. Participants were informed that their participation in this study would not affect their employment status. The study protocol complied with the Declaration of Helsinki and was approved by the Ethics Committee of Shandong Normal University (2023036).

### 2.2 Experimental design

**FIGURE 1:**
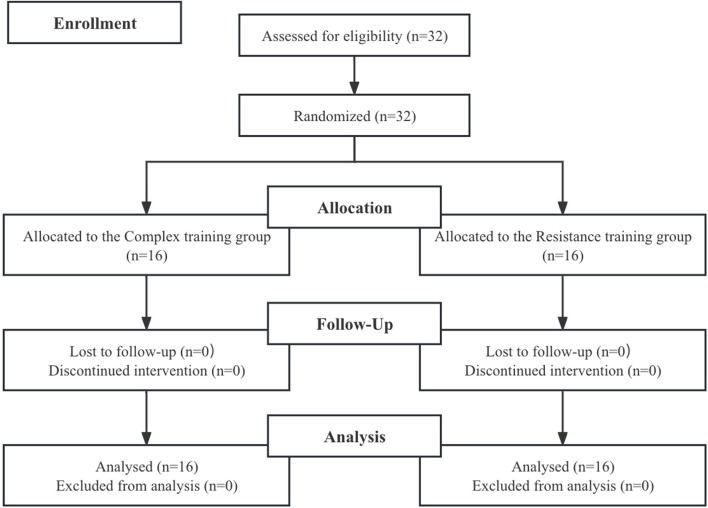
Flow chart of the progress through the phases of the study according to the CONSORT statements.

This study employed a non-randomized control trial to examine the comparative effects of two distinct training modalities. Participants were allocated into two groups based on team affiliation: the Resistance Training (RT) group (n = 16, age 18.93 ± 0.88 years) and the Complex Training (CT) group (n = 16, age 18.80 ± 0.68 years) (Table 1; Figure 1). An 8-week technical training regimen was systematically applied across all experimental protocols to ensure consistent progression and enable direct comparison between the CT and RT cohorts. Training sessions for both groups were scheduled concurrently on Mondays and Thursdays from 8:00 to 9:30 p.m. Evaluations to assess the impact of the interventions on futsal-related performance were conducted 1 week prior to and within 1 week following the conclusion of the training period. The testing conditions, including sequence, personnel, and location, remained uniform throughout the study. Pre- and post-intervention assessments included the Futsal Special Performance Test (FSPT), RSA, sprint decrement (Sdec), sprint times at 10-m (T10), 10–20-m (T10-20), and 20-m (T20), 1RM back squat (1RM BS), isometric mid-thigh pull (IMTP), and countermovement jump (CMJ).

**TABLE 1:** The descriptive characteristics of the participants.

	Age (years)	Height (cm)	Weight (kg)	Training experience (years)
RT (n = 16)	18.93 ± 0.88	177.20 ± 4.49	68.53 ± 4.00	5.60 ± 0.83
CT (n = 16)	18.80 ± 0.68	177.80 ± 3.30	66.93 ± 3.99	5.80 ± 0.86

### 2.3 Training programs

The characteristics of the CT and RT programs are detailed in Table 2, highlighting the progression and exercises used. To accommodate variations in participants’ physical condition, a flexible range of repetitions was set for each exercise, aiming for a consistent workload across participants. In the CT protocol, participants performed an RT exercise (Squat or Deadlift, four to six reps) followed by a plyometric exercise (Squat Jump or High pull, 10–12 reps) within each pair, with a 4-min rest between exercises and pairs.

**TABLE 2:** Resistance training and plyometric training program.

		Training content	Intensity			Sets* repetitions	Rest (min)
			The first stage (1–2weeks)	The second stage (3–4 weeks)	The third stage (5–8 weeks)		
CT	The No.1 session of every week	Squat + Squat Jump	75%1RM + ME	80%1RM + ME	85%1RM + ME	3* (4–6 + 10–12)	4
	No.2 session every week	Deadlift + High pull	75%1RM+50%1RM	80%1RM+50%1RM	85%1RM+50%1RM	3* (4–6 + 10–12)	4
RT	The No.1 session of every week	Squat	75%1RM	80%1RM	85%1RM	6* (6–10)	4
	No.2 session every week	Deadlift	75%1RM	80%1RM	85%1RM	6* (6–10)	4

Note: CT, complex training; RT, resistance training; 1RM, 1-repetition maximum; ME, maximal effort.

The RT group underwent an 8-week RT intervention, following the same schedule. Each session involved six sets of one RT exercise (Squat or Deadlift), with 6–10 reps per set and a 4-min rest between sets. Every 4 weeks, the 1RM for each exercise was assessed to adjust training intensity, based on Tomchuk (2010). If regular training sessions were missed, make-up sessions were scheduled for Saturday from 8:00 to 10:00 a.m.

Both groups began each session with a standardized 8–15 min warm-up, including low-intensity running, coordination exercises, dynamic movements, sprints, and dynamic lower-limb stretching. A standard 8–15 min cooldown, consisting of static stretching, concluded each session. Researchers specialized in strength training, conditioning, and physical fitness oversaw the development and monitoring of all training protocols.

#### 2.3.1 Futsal special performance test (FSPT)

**FIGURE 2:**
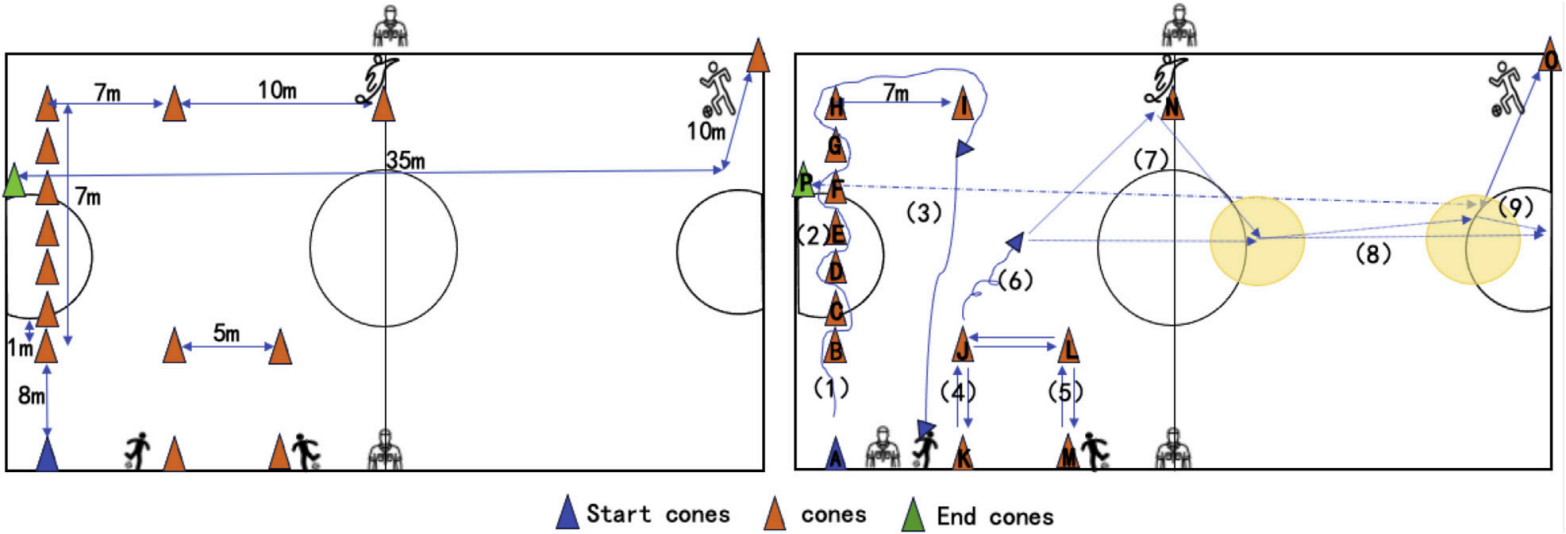
Schematic representation of Futsal Special Performance Test.

The performance and skills of futsal players were assessed through nine steps, including running with the ball, dribbling, turning, long and short passes, catching passes, performing wall passes, and fast running without the ball. The time of each step was measured, and the total time was calculated(s). After warm-up, participant was located behind the purple cone. In steps 1 and 2, participant runs with the ball and dribbles. Afterward, the participant turns to cone I, sends a long pass, and goes near cone J (step 3). After repetition, steps 4 and 5 include receiving and sending short pass, and participant rotates and dribbles; then, he performs a wall pass and shoots the ball to the goal. Finally, in step 9, the participant receives long pass and shoots to the goal, and subsequently goes to the final cone (Figure 2). Time was calculated by two referees, and average of their records was considered as total time. The penalty time was also recorded during the test, and performance time is obtained by adding penalty time to the total time. The test is carried out following the recommendations of Farhani et al. (Farhani et al., 2019). The interval between each trial was 1 min, and the best result of three times was taken for subsequent analysis. The ICC for this test was 0.88.

#### 2.3.2 RSA test

The test involved nine 20-m sprints with 25 s of active recovery between each repetition (jogging back to start within 20 s) to allow for 4–5 s of passive recovery (Torres-Torrelo et al., 2017). The times of the nine sprints taken by the athletes were recorded using the Polifemo Radio Light photocells from Microgate in Bolzano, Italy. The athletes started each sprint 1 m behind the timing lights and were verbally encouraged to run as fast as possible, and the RSAmean was calculated. Before the RSA, players performed a standardized warm-up consisting of a 5-min low-intensity run, three sets of 20-m progressive accelerations, and a maximum 10-m sprint with a 3-min break in between. The ICC was 0.87.

#### 2.3.3 10-m and 20-m sprint test

Sprint times were recorded for a distance of 20 m on an indoor running track (Buchheit and Mendez-Villanueva, 2014). Each participant completed two 20-m sprints with a 3-min break in between. Photocell timing gates from Polifemo Radio Light (Microgate, Bolzano, Italy) were placed at 0, 10, and 20 m to determine the time spent for 0–10 m (T10), 0–20 m (T20), and 10–20 m (T10-20). Participants adopted a standing start with the lead-off foot placed 1 m behind the first timing gate and gave their fullest effort to sprint. The best time for each interval (best T10, T20, and T10-20 of both trials) was retained for analysis. The warm-up included sets of 30-m sprints with a gradual acceleration. The intra-class correlation coefficients (ICC) were 0.91 for T10, 0.93 for T20 and 0.86 for T10–20.

#### 2.3.4 One-repetition maximum back squat

According to the protocol proposed by Tomchuk (2010) (Paz-Franco et al., 2017a), the back squat (BS) exercise was used to determine the maximal leg strength of each individual by a squat rack (Good Family, Shenzhen, China) To determine the maximum weight a participant can lift throughout the full range of motion (90° knee flexion), they first performed five to six repetitions with a relatively low weight (∼40% of their last 1RM test), followed by three to four repetitions with a larger weight (∼70% of their estimated 1RM). A single repetition was then performed with a load equivalent to 95% of the estimated 1RM. Afterward, the participant attempted a single repetition with the perceived 1RM load. When participants lifted the weight with the correct technique, they performed another attempt with an increase of 1.0–2.5 kg in weight. Failure was defined as a lift that did not reach the full range of motion on at least two trials with a 2-min break between trials. The test-retest reliability coefficient (ICC) for this test was 0.97 (Grgic et al., 2020).

#### 2.3.5 Isometric mid-thigh pull test

The lower limb isometric strength was assessed by the isometric mid-thigh pull (IMTP) test (Comfort et al., 2015a). Before testing, the midpoint between the knee and hip joints was marked to determine the mid-thigh position of each participant. The participants could choose their preferred hip and knee angles for the deadlift. The height of the barbell was adjusted to touch the mid-thigh. The participants could use a two-handed grip, a mixed grip, or a hooked grip to pull the barbell upward as hard and fast as possible for 6 s, using the peak force in 6s for analysis. To avoid precontraction, participants were required to relax before the command “GO!” The force-time curve for each trial was recorded by a force plate (Kistler 9281CA, KISTLER, Winterthur, Switzerland) at a sampling rate of 1,000 Hz (Comfort et al., 2015b). The interval between each trial was 1 min, and the best result of three times was taken for subsequent analysis. The ICC for this test was 0.85.

#### 2.3.6 Vertical-jump performance

The CMJ test was performed using the Ergo Jump Bosco System (Globus, Treviso, Italy) according to procedures proposed by Bosco et al. (Bosco et al., 1983). Jump height was determined based on flight time. Participants were requested to keep their bodies vertical throughout the jump, avoiding inappropriate lateral and frontal movements. The maximum vertical jump was performed from a standing position. The participants squatted to their self-selected depth and fully extended their knees on the ground. The interval between each trial was 1 min, and the best result of three times was taken for subsequent analysis. The hands of participants were placed on the hips during the jump to avoid any effect of arm-swing. The ICC for CMJ was 0.96.

### 2.4 Statistical analysis

Experimental data were processed by the IBM SPSS statistical software package (version 25.0, IBM, Chicago, IL, United States). All data were expressed as means and standard deviations (SD). The normality and homogeneity of variance were assessed by the Shapiro–Wilk and Levene’s Test, respectively. Outliers, defined as studentized residuals greater than three standard deviations from zero, were identified and removed. The significance level for all tests was set at *p* < 0.05. To examine the effects of the CT on the athlete’s performance in the futsal special performance test, RSA test, sprint test, and vertical-jump test, we first performed a two-way repeated-measure ANOVA (group × time). The dependent variables for each model were FSPT, RSAT, T10, T10–20, T20, 1RM BS, IMTP, and CMJ. The model factors were group, time, and their interactions. When significant interactions were observed, LSD *post hoc* corrections were performed to identify the location of significance. The intra-group effect size (ES) was also calculated using Hedge’s g formula (29). The threshold values for assessing the magnitude of the standardized effects were 0.20, 0.60, 1.20, 2.00 for small, moderate, large, and very large, respectively (Hopkins et al., 2009). The analysis also included calculating probabilities to determine if the true (yet unknown) differences fell below, matched, or exceeded the smallest significant difference or change, defined as 0.2 times the between-subject standard deviation (SD) (Cohen, 1988). We assessed the quantitative chances of observing better or worse effects through a qualitative scale: less than 1% indicates it is almost certainly not going to happen; 1%–5% is very unlikely; 5%–25% is unlikely; 25%–75% suggests it is possible; 75%–95% means it is likely; 95%–99% is very likely; and over 99% is almost certain. If the probability of achieving either beneficial or detrimental outcomes was greater than 5%, we classified the true difference as unclear (Hopkins et al., 2009). To estimate the sensitivity to change, we calculated the calculated the minimal detectable change (MDC = $\sqrt{2}$ x SEM x 1.96) (Sainani, 2017) and MDC% ((MDC/mean of pretest) x 100) (Lexell and Downham, 2005). Furthermore, we calculated Pearson’s correlation coefficients to explore the relationship between the percentage changes in all measured physical performance indicators. The significance of each correlation was classified using specific thresholds: below 0.1 as trivial; 0.1 to 0.3 as small; 0.3 to 0.5 as moderate; 0.5 to 0.7 as large; 0.7 to 0.9 as very large; and 0.9 to 1.0 as almost perfect (Hopkins et al., 2009). The relative reliability of the test was assessed using the intraclass correlation coefficient of the 1-way random-effects model with single measure ICC. The ICC of the FSPT, RSA, IMTP, and vertical jump tests was assessed by comparing the first two results obtained during the baseline test. A *p*-value of less than 0.05 was considered statistically significant for all tests.

## 3 Results

All the participants completed this study, and the data obtained from them were used in the analysis. All the data were normally distributed and homogeneity. At baseline, there were no significant differences between groups in any variable analyzed (*p* > 0.05). No significant correlation was observed between any measure and body weight for either CT or RT (Supplementary Table S1).

### 3.1 Futsal-specific performance test

After the 8-week training intervention, significant “time*group” interactions were observed for FSPT (*p* < 0.001). Intra-group comparisons revealed significant differences in the FSPT for the CT (*p* < 0.001, ES = 3.260) and RT (*p* < 0.001, ES = 1.079). Post-hoc analysis confirmed that the scores for FSPT (*p* < 0.05) was higher after the CT intervention than before and after the RT intervention. The CT showed a very likely effect in the FSPT, while the RT presented an unclear effect (Table 3).

**TABLE 3:** Assessment results of the CT and RT groups before and after 8 weeks of training.

	MDC (%)	CT group					RT group				
		Pre	Post	Δ (%) (90% CI)	Percent changes of better/Trivial/Worse effect	ES	Pre	Post	Δ (%) (90% CI)	ES	Percent changes of better/Trivial/Worse effect
FSPT (s)	3.63	31.43 ± 1.78	28.01 ± 1.56∗#	−10.84 (−12.13, −9.56)	100/0/0 Most likely positive	3.26	31.47 ± 2.72	30.35 ± 2.44∗	−3.45 (−4.75, −2.16)	1.08	80/13.33/6.66 Unclear
RSAT_mean_ (s)	1.27	3.22 ± 0.09	3.15 ± 0.090∗	−1.96 (−3.25, −0.67)	73.33/6.66/6.66 Unclear	0.68	3.21 ± 0.06	3.17 ± 0.09	−1.23 (−2.53, 0.07)	0.41	26.66/60/6.66 Unclear
Sdec (%)	6.62	4.56 ± 0.65	4.17 ± 0.34∗	−7.64 (-12.10, −3.17)	66.66/26.66/6.66 Unclear	0.69	4.54 ± 0.56	4.38 ± 0.54	−3.49 (−5.66, −1.32)	0.67	33.33/60/6.66 Unclear
T10 (s)	1.41	1.76 ± 0.04	1.66 ± 0.06∗#	−5.18 (−6.75, −3.61)	100/0/0 Most likely positive	1.40	1.75 ± 0.06	1.74 ± 0.07	−0.08 (−1.11, 0.95)	0.05	40/33.33/26.66 Unclear
T10-20 (s)	1.79	1.24 ± 0.04	1.22 ± 0.03	−0.90 (−2.80, 1.01)	53.33/6.66/40 Unclear	0.23	1.24 ± 0.04	1.22 ± 0.03	−0.90 (-2.84, 1.03)	0.23	66.66/6.66/26.66Unclear
T20 (s)	0.96	2.95 ± 0.64	2.90 ± 0.66∗	−1.82 (−2.94, −0.71)	73.33/13.33/13.33 Unclear	−0.69	2.95 ± 0.05	2.90 ± 0.08*	−1.67 (−3.20, −0.13)	−0.48	66.66/6.66/26.66Unclear
1RM BS (kg)	3.10	125.53 ± 9.13	132.53 ± 8.52∗	5.67 (4.35, 7.00)	100/0/0 Most likely positive	1.90	125.53 ± 9.13	130.33 ± 5.86∗	4.48 (2.19, 6.76)	0.83	80/13.33/6.66Unclear
IMTP (N)	2.64	1899.61 ± 102.70	2043.06 ± 55.31∗#	7.80 (5.28, 10.31)	100/0/0 Most likely positive	1.47	1901.87 ± 99.09	1994.55 ± 48.82*	5.12 (2.56, 7.67)	0.94	80/13.33/6.66Unclear
CMJ (cm)	6.62	35.27 ± 2.86	38.66 ± 1.33∗#	10.23 (6.38, 14.09)	100/0/0 Most likely positive	1.37	35.75 ± 1.21	37.33 ± 1.14	4.46 (3.17, 5.75)	0.29	86.66/0/3.33Unclear

Note: MDC: minimal detectable change; FSAT: futsal special performance test; RSATmean: mean sprint time of the nine sprints; Sdec: percent sprint decrement for the nine sprints; T10:10-m sprint time; T10–20: 10–20-m sprint time; T20: 20-m sprint time; 1RM BS: One-repetition maximum back squat; IMTP: Isometric Mid-thigh Pull; CMJ, countermovement jump. *Statistically significant difference between pre-and post-test, *p* < 0.05; # significant difference between the CT, and TT, groups in the post-test, *p* < 0.05. Δ: Percentage change; ES: Effect size Hedge´s *g*; CI: confidence interval.

### 3.2 RSA test

After the 8-week training intervention, no significant “time*group” interactions were observed for RSATmean and Sdec (*p* > 0.05). Intra-group comparisons revealed significant differences in the RSATmean (*p* < 0.05, ES = 0.678) and Sdec (*p* < 0.05, ES = 0.687) only in the CT. The CT and RT were showed unclear effect in the RSATmean and Sdec (Table 3).

### 3.3 Sprint test

After the 8-week training intervention, significant “time*group” interactions were observed for T10 (*p* < 0.001). Intra-group analysis showed significant improvements in the T10 (*p* < 0.05, ES = 1.400) and T20 (*p* < 0.05, ES = 0.687) only in the CT. Post-hoc analysis confirmed that the scores for T10 (*p* < 0.05) was higher after the CT intervention than before and after the RT intervention. The CT showed a very likely effect in the T10, while the RT presented an unclear effect. The CT and RT were showed an unclear effect in the T20 and T10-20 (Table 3). Intra-group comparisons revealed significant differences in the FSPT for the T10 (*p* < 0.001, ES = 3.260) and RT (*p* < 0.001, ES = 1.079).

### 3.4 One-repetition maximum back squat

After the 8-week training intervention, no significant “time*group” interaction was observed for 1RM BS (*p* > 0.05). Intra-group comparisons revealed significant differences in the 1RM BS for the CT (*p* < 0.05, ES = 1.902) and RT (*p* < 0.05, ES = 0.826). The CT showed a very likely effect in the 1RM BS, while the RT presented an unclear effect (Table 3).

### 3.5 Isometric mid-thigh pull test

After the 8-week training intervention, no significant “time*group” interaction was observed for IMTP (*p* > 0.05). Intra-group comparisons revealed significant differences in the IMTP for the CT (*p* < 0.05, ES = 1.467) and RT (*p* < 0.05, ES = 0.944). The CT showed a very likely effect in the IMTP, while the RT presented an unclear effect (Table 3).

### 3.6 Vertical-jump performance

After the 8-week training intervention, significant “time*group” interactions were observed for CMJ (*p* < 0.001). Intra-group comparisons revealed significant differences in the CMJ for the CT (*p* < 0.001, ES = 1.372) and RT (*p* < 0.001, ES = 0.294). Post-hoc analysis confirmed that the scores for CMJ (*p* < 0.05) was higher after the CT intervention than before and after the RT intervention. The CT showed a very likely effect in the CMJ, while the RT presented an unclear effect (Table 3).

### 3.7 Correlations between changes in physical performance variables.

**FIGURE 3:**
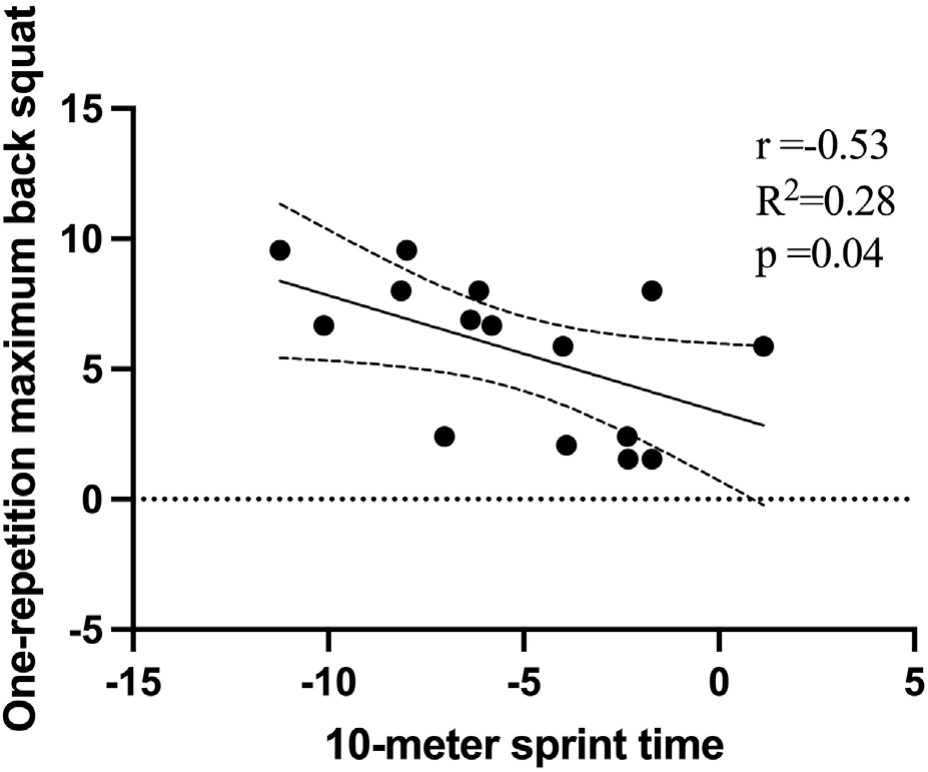
Relationship between percentage change in one-repetition maximum back squat and percentage change in 10-m sprint time performance.

**FIGURE 4:**
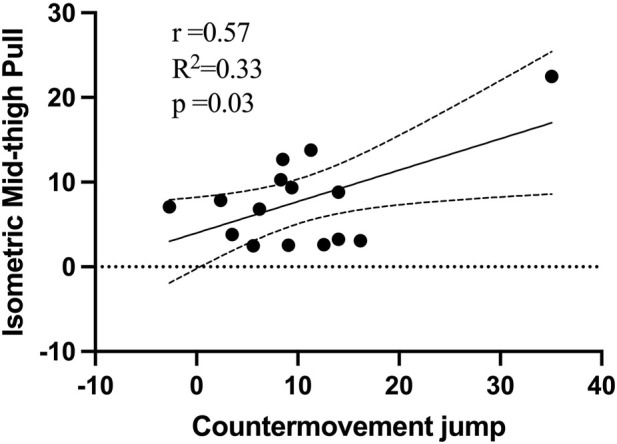
Relationship between percentage change in Isometric Mid-thigh Pull and percentage change in countermovement jump performance.

A negative correlation was observed between the percentage change in T10 and the 1RM BS (*p* = 0.04, r = −0.53), as shown in Figure 3, for the CT. And a positive correlation was observed between the percentage change in CMJ and the IMTP (*p* = 0.03, r = 0.57), as shown in Figure 4, for the CT. No significant correlations were observed for the RT.

## 4 Discussion

This study aimed to compare the effects of CT vs. RT on physical and technical performance in amateur futsal players. The results demonstrated that CT improved FSPT, T10, IMTP, and CMJ more than RT. On the other hand, no differences between groups were observed on RSA, Sdec, T10–20, T20, and 1RM BS. Overall, our study suggests that CT emerges as an effective method for augmenting performance in amateur futsal players.

### 4.1 Futsal-specific performance test

FSPT primarily assesses skills such as passing, dribbling, and shooting, in addition to abilities like speed and agility, which are crucial in futsal. Previous research has demonstrated the FSPT as a reliable measure for evaluating skill-related aspects in futsal (Farhani et al., 2019). These findings of this study suggest that CT could be an effective approach to enhance sports-related skills in futsal, especially in augmenting speed and agility. This is in line with our hypothesis that CT may be more effective than RT in enhancing movements involved in the stretch-shortening cycle function (Markovic and Mikulic, 2010). The CT regimen included exercises with both high loads (above 75% 1RM) to enhance the strength-speed segment and low loads (below 50% 1RM) to optimize the maximum velocity segment of the force-velocity curve (Haff and Nimphius, 2012). This approach aims to simultaneously improve maximal and explosive strength, thereby maximizing sport-specific performance. In contrast to CT, the higher loads employed in RT typically lead to slower muscle contractions, potentially making it less effective for enhancing explosive strength. Paz-Franco investigated the effects of different resistance training (RT) frequencies on professional futsal players over a 6-week period concluding that one or 2 RT sessions per week alongside regular futsal training significantly enhance physical performance, suggesting that RT once every second week is insufficient for improving physical fitness in professional futsal players (Paz-Franco et al., 2017b). Marques conducted over an 8-week in-season period with elite futsal players focused on evaluating the effects of a resistance training (RT) program on strength and power performance, observing that higher recovery quality could predict lower perceived exertion in training sessions (Marques et al., 2022). The study conducted over an 8-week in-season period with elite futsal players focused on evaluating the effects of a resistance training (RT) program on strength and power performance. It also examined the relationship between session perceived exertion (sRPE), total quality recovery (TQR), and the volume load of RT. Significant improvements were observed in isometric hip adduction strength (IHAS), with small yet significant enhancements in peak power and countermovement jump (CMJ). While our study utilized indirect measures of SSC function, future research would benefit from the inclusion of direct SSC assessments to more accurately evaluate the impact of training modalities on this crucial aspect of futsal athleticism. However, most changes did not exceed the minimal detectable change (MDC), suggesting that low-volume, low-to-moderate load RT might not sufficiently stimulate dynamic strength and power gains in elite players, although it can enhance isometric strength. Additionally, the study found a significant negative correlation between TQR and sRPE, indicating that higher recovery quality could predict lower perceived exertion in training sessions.

Consequently, CT might be a more effective training methodology for enhancing sport-specific performance, particularly during pre-season strength training.

### 4.2 Repeated sprint ability test

Previous research suggests that RSA is influenced by greater muscle power output (Okuno et al., 2013), indicating that CT could potentially improve RSA. Our findings partially corroborate this, which the result show that CT led to a significant reduction in RSATmean despite no significant group difference between CT and RT. The improved velocity in 10 m sprinting likely explains CT’s effectiveness in improving the RSATmean. While sprinting velocity, a critical factor of RSA, has shown significant improvement through CT, RSA is also constrained by factors such as energy supply capacity, hydrogen ion (H+) removal, and muscle activity (Bishop et al., 2011). CT primarily focuses on neuromuscular aspects minimally impacting the metabolic system which explains the absence of significant differences observed between CT and RT. Torres-Torrelo found that a 6-week training intervention combining resistance training (RT) with loaded change of direction (CD) exercises significantly improved muscle strength and repeated sprint ability (RSA) in futsal players compared to RT alone and a control group with no training changes. The combined RT and CD group exhibited greater enhancements in RSA mean sprint times and ground contact time across multiple sprints, indicating the efficacy of combining RT with dynamic, sport-specific movements for futsal performance enhancement (Torres-Torrelo et al., 2018). Nevertheless, our results suggest that CT could be an effective approach to enhance RSA, warranting further investigation.

### 4.3 Sprinting performance

Both CT and RT demonstrated significant improvements in the T20, with no significant differences between the groups. However, CT showed a significant improvement in T10 compared to RT, while no significant difference in T10-20 in either group. In futsal, 10 m sprinting is a more relevant ability, as the majority of sprinting distances are between 10 and 20 m (Naser et al., 2017). Moreover, improvement in T10 may contribute to the improvement in RSA and FSPT, which partly explains the significant differences in CT instead of RT. Since sprint performance is heavily influenced by maximal strength, and both CT and RT involve high-intensity resistance training, both may have an effect on 20-m sprint performance. The study of Torres-Torrelo examined the impact of a 6-week training program on futsal players, indicating significant improvements in countermovement jump, ball throwing distance, and 30 m sprint performance with no significant changes observed in the control group. This suggests that incorporating resistance training with change of direction exercises can positively affect explosive power and speed in futsal players (Torres-Torrelo et al., 2017). The 10-m sprint, more dependent on the rate of force development (Naser et al., 2017), is significantly impacted by the plyometric training in CT. This might be re reason why CT effectively increases the rate of force development in the muscles, thus leading to more significant improvements in T10.

### 4.4 Strength and power test

1RM BS and IMTP were significantly increased after both CT and RT for IMTP, CT achieved a more significant improvement. Previous studies suggest that IMTP is more effective in detecting the positive effects of RSA (Bender et al., 2018), which is in line with the improvement of RSATmean in CT. Additionally, the peak force generation was significantly increased in IMTP, possibly an underlying mechanism of enhanced RSA due to improved efficiency of skeletal muscle contraction (Haider and Folland, 2014). Marques observed significant improvements in physical performance, including countermovement jump height, *t*-Test time, kicking ball speed, and maximum dynamic strength, indicating that such a training program can effectively enhance various performance parameters in young futsal players without prior RT experience (Marques et al., 2019). Overall, CT has more increased complexity and specificity than RT and may thus have more central nervous system stimulation, thereby promoting better neural adaptations such as greater intermuscular coordination and the synchronization of muscle fiber recruitment. In other words, CT is a more effective method to develop sports-specific ability and RSA compared to RT.

Importantly, the results of this study do not conclusively demonstrate the superiority of CT over RT in enhancing overall athletic performance. Both training programs contributed to performance improvements, with CT showing a trend for greater gains and effect sizes in some variables but not significantly different in RSA, Sdec, T10–20, T20, and 1RM BS. Our findings underscore the effectiveness of both CT and RT’s effectiveness in augmenting amateur futsal players’ athletic performance. While RT can provide the foundational strength necessary for athletic performance, CT’s incorporation of plyometric exercises can further enhance power and technical performance, which is critical for futsal. Implementing periodization strategies, where the focus of training shifts between CT and RT across different phases of the season, can help optimize performance peaks and recovery. For instance, a pre-season focus on RT to build strength followed by a mid-season emphasis on CT to sharpen power and technical performance.

### 4.5 Correlations between Changes in Physical Performance Variables

In research examining the impact of CT, a notable negative correlation was found between gains in 10-m sprint times and 1RM BS performance, indicated by a correlation coefficient of −0.53. Conversely, a positive correlation emerged between lower limb power, as measured by the CMJ, and lower limb isometric strength, quantified through the IMTP), with a correlation coefficient of 0.57. These findings align with outcomes reported in previous studies after implementing CT programs (Mason et al., 2021; Liu et al., 2022; Qiao et al., 2022). For instance, Comfort and others, in their study with elite 17-year-old soccer players, identified a similarly strong negative correlation between maximal strength and 20-m sprint times, with a coefficient of −0.64 (Comfort et al., 2014). Additionally, a substantial correlation between IMTP and CMJ was documented in another investigation, reinforcing the theory that augmentations in lower-body strength positively influence sprint performance and lower limb power (Mason et al., 2021). Therefore, the CT approach, which emphasizes high-load resistance training combined with plyometric exercises, is increasingly recognized as an effective strategy for enhancing sprint performance and the power of the lower limbs.

The limitations of this study are multifaceted. Primarily, the findings presented herein necessitate verification through future investigations to bolster their validity. A significant limitation to acknowledge is the power analysis; although it adhered to established best practices. This acknowledgment serves not only to uphold transparency but also to guide and improve the methodological rigor in future research endeavors. Additionally, we recognize the omission of direct stretch-shortening cycle (SSC) measurements as a constraint in our evaluation. The SSC is pivotal to many futsal movements, and its assessment could provide a deeper understanding of the impact of CT on performance. This omission points to the need for a more comprehensive battery of tests in future studies, which should include SSC measures to offer a more complete picture of the interventions’ effectiveness. The current study’s focus on RSA may not fully represent the broad spectrum of functional adaptations that occur as a result of CT. Therefore, it is imperative to extend future assessments to encompass a wider range of performance indicators, including those that more directly reflect on-court demands. We also suggest that subsequent research should investigate the dose-response relationship between CT and performance outcomes in a more detailed manner. Employing a variety of assessments at multiple points throughout the intervention would yield valuable insights and enable a fine-tuning of training protocols to optimize athlete performance enhancements. Longitudinal studies are warranted to understand the long-term effects and sustainability of the benefits of CT. These should explore variations in CT program parameters, to identify the enduring effects of the training and to optimize session frequency and intensity for peak athletic outcomes.

## 5 Conclusion

The current study showed a tendency for greater physical and technical performance gains following CT compared to RT in amateur futsal players. Although both interventions may be valuable for improving specific performance parameters in amateur futsal players, CT may offer more advantages over RT in improving physical and technical performance. These findings support tailored strength and conditioning training programs for futsal players to optimize physical and technical performance.

## Data Availability

The original contributions presented in the study are included in the article/Supplementary Material, further inquiries can be directed to the corresponding author.
