# Supplementary material for: A comparative study of 8-week complex training and resistance training on athletic performance of amateur futsal players
Source: Front Physiol. 2024 Apr 26;15:1360440. doi: 10.3389/fphys.2024.1360440 (PMC11088241; doi:10.3389/fphys.2024.1360440)
Supplement: Supplementary file 1 [file Table1.DOCX]

|  | Pro-Weight (kg) | Post-Weight (kg) | FSPT (s) | RSAT_mean_ (s) | Sdec (%) | T10 (s) | T10-20 (s) | T20 (s) | 1RM BS (kg) | IMTP (N) | CMJ (cm) |
| --- | --- | --- | --- | --- | --- | --- | --- | --- | --- | --- | --- |
| RT(n=16) | 68.53±4.00 | 68.60±4.05 | r = 0.172  p = 0.063 | r = 0.159  p = 0.080 | r = -0.158  p = 0.082 | r = -0.160  p = 0.075 | r = -0.158  p = 0.080 | r = -0.141  p = 0.114 | r = 0.170  p = 0.066 | r = 0.175  p = 0.056 | r = 0.174  p = 0.055 |
| CT(n=16) | 66.93±3.99 | 67.03±4.02 | r = 0.169  p = 0.066 | r = 0.152  p = 0.086 | r = -0.152  p = 0.084 | r =-0.169  p = 0.061 | r = -0.161  p = 0.076 | r = -0.148  p = 0.105 | r = 0.172  p = 0.062 | r = 0.169  p = 0.060 | r = 0.172  p = 0.054 |

**Table S1. Relationship between percentage change in body weight and percentage change in each index.**
